# From fever to anti-malarial: the treatment-seeking process in rural Senegal

**DOI:** 10.1186/1475-2875-9-333

**Published:** 2010-11-22

**Authors:** Lucy A Smith, Jane Bruce, Lamine Gueye, Anthony Helou, Rodio Diallo, Babacar Gueye, Caroline Jones, Jayne Webster

**Affiliations:** 1 Disease Control & Vector Biology Unit, Department of Infectious & Tropical Diseases, London School of Hygiene & Tropical Medicine, Keppel Street, London. WC1E 7HT, UK; 2 IntraHealth International, Lot C & E, Sacré-Coeur Pyrotechnie, Dakar, Senegal; 3 Pfizer Canada Inc., Montreal, Canada

## Abstract

**Background:**

Currently less than 15% of children under five with fever receive recommended artemisinin-combination therapy (ACT), far short of the Roll Back Malaria target of 80%. To understand why coverage remains low, it is necessary to examine the treatment pathway from a child getting fever to receiving appropriate treatment and to identify critical blockages. This paper presents the application of such a diagnostic approach to the coverage of prompt and effective treatment of children with fever in rural Senegal.

**Methods:**

A two-stage cluster sample household survey was conducted in August 2008 in Tambacounda, Senegal, to investigate treatment behaviour for children under five with fever in the previous two weeks. The treatment pathway was divided in to five key steps; the proportion of all febrile children reaching each step was calculated. Results were stratified by sector of provider (public, community, and retail). Logistic regression was used to determine predictors of treatment seeking.

**Results:**

Overall 61.6% (188) of caretakers sought any advice or treatment and 40.3% (123) sought any treatment promptly within 48 hours. Over 70% of children taken to any provider with fever did not receive an anti-malarial. The proportion of febrile children receiving ACT within 48 hours was 6.2% (19) from any source; inclusion of correct dose and duration reduced this to 1.3%. The proportion of febrile children receiving ACT within 48 hours (not including dose & duration) was 3.0% (9) from a public provider, 3.0% (9) from a community source and 0.3% (1) from the retail sector. Inclusion of confirmed diagnosis within the public sector treatment pathway as per national policy increases the proportion of children receiving appropriate treatment with ACT in this sector from 9.4% (9/96) to an estimated 20.0% (9/45).

**Conclusions:**

Process analysis of the treatment pathway for febrile children must be stratified by sector of treatment-seeking. In Tambacounda, Senegal, interventions are needed to increase prompt care-seeking for fever, improve uptake of rapid diagnostic tests at the public and community levels and increase correct treatment of parasite-positive patients with ACT. Limited impact will be achieved if interventions to improve prompt and effective treatment target only one step in the treatment pathway in any sector.

## Background

Prompt and effective case management is a key component of global strategies to reduce the burden of malaria. The Roll Back Malaria Partnership (RBM) has set the target for 2010 that "80% of malaria patients are diagnosed and treated with effective anti-malarial medicines, e.g. artemisinin-based combination therapies (ACT), within one day of the onset of illness" [1]. Available data on progress towards this goal suggest that the mean proportion of children under five years of age with a fever that were treated with an anti-malarial drug in sub-Saharan Africa between 2007 and 2008 was 32% (varying across countries from 6% to 57%); less than 15% of children were given an ACT (range: 3% to 25%) [2]. These data do not include a measure of the promptness of treatment. Therefore, considering the delays in treatment-seeking reported elsewhere [3], it is likely that the proportion of children receiving an appropriate anti-malarial drug within one day of the onset of illness will be even lower.

Alongside the shift in national treatment polices to the use of more expensive forms of ACT, the policy process has recently changed to include parasitological diagnosis rather than presumptively treating all fevers as malaria [4]. This is particularly the case in the public sector [5,6], however increasingly discussions are also moving towards introducing use of rapid diagnostic tests (RDTs) by community health workers [7,8]. Thus, it is important to consider diagnosis-confirmed cases of malaria, not just any febrile illness when assessing the proportion of malaria cases receiving prompt and effective treatment.

In order to understand why prompt and effective treatment of malaria (or fever) remains low it is necessary to examine the treatment pathway, comprising both treatment-seeking and the delivery process. Critical blockages in this treatment pathway may be identified by assessing the probability of a child moving from one step to the next, from getting a fever to receiving appropriate treatment [9]. Once critical blockages in the treatment pathway have been identified it may be possible to design new, or re-focus current interventions to move more rapidly towards the RBM target. If effective treatment of malaria is to be improved for children across socioeconomic groups, all sectors involved in the delivery of anti-malarials must be considered in this 'diagnostic' manner. Such an approach will enable the targeting of interventions to increase effective treatment at all sources that the caretakers of children are utilizing [10].

This paper presents the application of such a diagnostic approach to the coverage of prompt and effective treatment for febrile children in rural Senegal, assessing the critical steps at which children exit from the treatment pathway, stratified according to source of first advice or treatment.

## Methods

### Study area and background

Tambacounda is the largest region in Senegal, located in the south-east of the country and bordered by The Gambia, Guinea and Mali. The rainy season is from June to October and the majority of malaria transmission occurs between July and November [11]. Despite recent trends showing a decrease in the proportion of morbidity and mortality due to malaria in Senegal, there is still substantial regional variation. In the Tambacounda region malaria accounts for 13.5% of all outpatient consultations and 22.4% of mortality [12] and Tambacounda has the highest parasite prevalence in children under five of 23.4% compared to the national figure of 5.7% [13].

The population of Tambacounda is widely dispersed and access to health care is often limited. The health system in Senegal is structured to have community outreach services at the village level through health huts (*cases de santé*), community health workers (*agent de santé communautaire*, ASC) and community health volunteers (*relais communautaire*, RC). However, in certain parts of the country these community level services are no longer functional. The international non-governmental organization IntraHealth is working to rehabilitate 24 health huts across Tambacounda, Koumpentoum and Maka Coulibantang districts. This rehabilitation was undertaken as part of an intervention to improve prompt and effective treatment of fever in children under five years with the national first-line anti-malarial, the ACT artesunate-amodiaquine (AS-AQ). The overall goals of the intervention are in alignment with the National Malaria Control Programme (*Programme National de Lutte contre le Paludisme*, PNLP) strategic plan to ensure correct treatment of 80% of malaria cases at all levels of the health system [14]. Each health hut serves its own village and four additional 'polarised villages' (*villages polarisés*). Prior to the final design and implementation of the intervention, a household survey of treatment practices for children under five years with fever was conducted between August 22^nd ^and September 2^nd ^2008.

### Household survey design & implementation

The household survey followed a two-stage cluster sample design. The sampling frame for the survey covers all 110 villages that are served by the 24 intervention health huts in the districts of Tambacounda, Koumpentoum and Maka Coulibantang, which have an approximate total population of 37,770. Each village represented a cluster and clusters were sampled proportional to population size.

To estimate the proportion of children under five years with fever in the last two weeks who received anti-malarial drugs within 24 hours with 6% precision and assuming a design effect of 2, a non-response rate of 10%, and the percentage of children under 5 years in the population as 17.5%, a sample size of 240 was required [15]. To achieve this sample size, 40 clusters of 6 children under five years with fever in the last two weeks were sampled.

Households were selected within each cluster using an adapted form of the expanded programme of immunisation (EPI) cluster survey method [16]. A household was defined as all those eating from the same cooking pot [13]. Since the aim of this survey was to investigate the treatment practices for children under five years with fever, sampling of households was restricted to those with a child under five who had had fever in the past two weeks; no additional symptoms were specified in the inclusion criteria. Two selection questions were asked at each household: (i) Is there a woman present in this household with at least one child less than five years of age? (ii) Has this child (these children) had fever at any time in the last two weeks? Interviews were only continued with the child's caretaker where the answers to both of the selection questions were positive.

The survey tool included structured questions on respondent and household characteristics, details of the treatment process for children under five with fever (including source and timing of treatment, reported dose and duration of anti-malarials), and malaria-related knowledge.

### Data analysis

Data were double-entered and validated in EpiData version 3.1 [17]. Stata 10.0 [18] was used for data processing and analysis. All analyses accounted for the design of the survey, adjusting for clusters. The survey design was self-weighting.

The treatment pathway was divided into five key steps and the proportion of children reaching each step calculated. The total number of febrile children was used as the denominator for all steps: (i) child had fever in the previous two weeks (denominator); (ii) child was taken for treatment or advice; (iii) care was sought within 48 hours; (iv) child received an anti-malarial; (v) child received an ACT. Prompt treatment is considered to be within 48 hours, defined as the day that the fever starts or the next day; the reason being that it is difficult to be sure on the precise number of hours and it is likely that 'the next day' will be more than 24 hours. The source of first advice or treatment was categorized to: community-based delivery point (defined as a health hut, ASC or RC); public health facility (defined as a health post or health centre; none of the caretakers reported first seeking care at a public hospital); or retail delivery point.

Traditional healers and friends/relatives were excluded from this categorization since we were interested in places where allopathic anti-malarial drugs could be given. Steps ii-v were calculated for treatment from any source, and from community, public and retail sector sources.

Principal components analysis (PCA) was used to assign each household a wealth index score based on household characteristics and ownership of assets [19]. The wealth index score was then used to classify households into three socioeconomic groups (tertiles). The variables included in the PCA were type of roof material, type of floor material, and household ownership of the following assets: solar panel, radio, television, telephone, mobile, fridge, bed, kettle, bucket, battery, sleeping mat, plough, horse, cow, goat, donkey, bicycle, motorbike, car, oxcart, horse and cart, donkey and cart. The proportion of variation explained by the first principle component was 17.7%.

Univariable analyses of potential predictors of seeking any advice or treatment for a febrile child, and the source of first advice or treatment were explored using a logistic regression model; adjusted Wald tests were used to assess the contribution of each factor to the model. Potential predictors with a p-value of less than 0.1 were included in multivariable models for each of the outcome measures to ascertain which predictors remained associated with the outcome when adjusted for other factors.

### Modelling the impact of rapid diagnostic tests (RDTs)

The survey was designed to assess access to prompt effective treatment and therefore used the Roll Back Malaria (RBM) access indicator as an outcome measure [20]. The Senegalese PNLP strategic plan states that all childhood fevers should be treated with AS-AQ within 24/48 hours of onset. However, at the time of the survey, community-based diagnosis of malaria was based on symptoms alone (i.e. presumptive), whereas the policy stated that in public sector health facilities confirmatory diagnostic tests should be employed [14]. The RBM access indicator does not address the use of diagnostics in the treatment process and therefore no questions relating to diagnostics were included in the survey. As such, it was not possible to include empirical data on confirmed diagnosis in the algorithm for assessing appropriate treatment. To overcome this limitation we used routine data to model the impact of including RDT diagnosis in the treatment pathway assessments for the public sector.

For those seeking treatment from a public sector provider two additional steps were inserted into the treatment pathway. These steps were: the proportion receiving diagnosis with an RDT; and amongst those tested with an RDT, the proportion parasite positive. According to the most recent data from the PNLP, the proportion of patients with fever attending a formal public health facility in the Tambacounda region that received an RDT in 2008 was 78%; the proportion of RDTs conducted that were positive for malaria in Tambacounda was 60% [12]. Thus, the denominator used for assessing appropriate treatment in the public sector where diagnosis should be RDT-confirmed was the expected number of children that would have confirmed malaria. This assumes that the decision not to administer an RDT to 22% of children with fever was based on sound clinical judgement of alternative diagnoses for these children rather than missed malaria cases.

### Ethical approval

Ethical approval for the study was granted by the ethics committees of the *Conseil National de Recherche en Santé *(CNRS), Dakar, Senegal and London School of Hygiene & Tropical Medicine, London, England.

## Results

Interviews were completed with 240 caretakers of children under five years of age with fever in the previous two weeks. As nearly a quarter of households had more than one child with a fever in the last two weeks a total of 305 febrile children were included in the survey sample. The mean number of febrile children under five years per household was 1.27 (range: 1 to 5 children). Since the majority of households with multiple febrile children under five years had two febrile children, potential clustering of treatment seeking outcomes was explored within these pairs of children. No agreement within child pairs for the outcome of receiving ACT within 48 hours was observed (correlation coefficient: 0.2344); therefore, the analysis was not adjusted for clustering at the household level. Household size varied from 3 to 39 people, with a mean of 11.0 ± 5.2. A total of 2,652 people lived in the households interviewed.

### Prompt treatment-seeking across sectors

Overall 61.6% (188) of caretakers sought any advice or treatment and 40.3% (123) sought any treatment promptly within 48 hours. Of the 188 children with fever for whom any advice or treatment was sought, the greatest number were taken to a health post (41.5%), the lowest level public health facility; approximately equal numbers were taken to a health hut or community volunteer (12.7%) or the retail sector (13.8%); and 9.6% were taken to a public sector health centre. The remaining children were taken to an 'other' source (including 13.8% to a traditional healer, and 4.8% to a friend or relative or that already had drugs in the house).

The proportion of all children with fever that accessed the community or retail sectors was low (7.8% and 8.5%, respectively); however those who did, did so promptly with 83.3% and 80.8% accessing these delivery points within 48 hours, respectively. A lower proportion of the febrile children accessing public sector delivery points did so promptly (63.2%). However, the difference in proportion promptly accessing the community (p = 0.12) or retail (p = 0.34) sector versus the proportion promptly accessing the public sector was not statistically significant. It is not unexpected that the community sector is the least accessed at the time of this survey as this was before the implementation of the health hut rehabilitation intervention.

### Predictors of seeking any advice or treatment

Univariable logistic regression suggests that children of higher socioeconomic status are more likely to seek advice or treatment from any source, including traditional healers and friends or family, with those in the highest socioeconomic third twice as likely to seek any advice or treatment than those in the lowest third (lowest tertile: referent; middle tertile: OR: 1.86; 95%CI: 0.98, 3.56; highest tertile: OR: 2.06; 95%CI: 1.07, 3.98; p = 0.05) (see Table 1). No other predictors were significantly associated with seeking treatment from any source, thus it was not appropriate to construct a multivariable model.

**Table 1:** Univariable logistic regression of the odds for seeking any advice or treatment for a child under five years with fever from any source.

Variable	N	%	OR	95% CI	P value
Child's age					0.9
< 1 year	74	62.0	1.00		
1-4 years	234	61.5	0.98	0.56, 1.73	

Child's gender					0.2
Male	154	58.4	1.00		
Female	151	64.9	1.31	0.83, 2.08	

Mother's education					0.1
None	183	57.4	1.00		
Any	122	68.0	1.58	0.91, 2.75	

Mother's age					0.3
< 20 years	56	71.4	1.00		
20-29 years	120	64.2	0.72	0.31, 1.66	
30-39 years	93	60.2	0.61	0.26, 1.39	
> 40 years	36	41.7	0.29	0.08, 1.01	

No. wives in HH					0.1
One or less	208	64.9	1.00		
Two or more	97	54.6	0.65	0.36, 1.17	

Household size					0.9
< 10 members	112	61.6	1.00		
10-14 members	129	62.8	1.05	0.54, 2.05	
15 or more members	64	59.4	0.91	0.45, 1.86	

No. U5s with fever in HH					1.0
One	183	61.7	1.00		
Two or more	122	61.5	0.99	0.51, 1.91	

Socioeconomic status*					0.05
Lowest tertile	98	50.0	1.00		
Middle tertile	106	65.1	1.86	0.98, 3.56	
Highest tertile	95	67.4	2.06	1.07, 3.98	

Seen malaria information in last year					0.7
No	127	63.0	1.00		
Yes	178	60.7	0.91	0.56, 1.47	

* 6 missing values for socioeconomic status

### Predictors of where initial advice or treatment was sought

Logistic regression was used to explore the predictors of seeking first advice or treatment at (i) a community-based delivery point; (ii) a public health facility; and (iii) a retail sector delivery point. Traditional healers and family or friends were excluded from this categorisation (n = 42). The factors influencing treatment-seeking from the different categories of providers varied. For example, the univariable analyses (Table 2) suggested that the child's age and socioeconomic status were univariable predictors of first seeking care in the formal public health sector. The child's age and sex, and the number of wives of the head of household were predictors of first seeking care from a community-based source. Child's age and household size were univariable predictors of first seeking care for a child under five with fever from a retail outlet.

**Table 2:** Univariable logistic regression models of the odds for seeking any advice or treatment for a child under five years with fever from the public sector, community sector or retail sector.

		First seek treatment in public sector				First seek treatment in community sector				First seek treatment in retail sector			
**Variable**	**N**	**%**	**OR**	**95% CI**	**P value**	**%**	**OR**	**95% CI**	**P value**	**%**	**OR**	**95% CI**	**P value**

Child's age					0.05				0.09				0.06
< 1 year	74	42.3	1.00			2.8	1.00			1.4	1.00		
1-4 years	234	28.2	0.54	0.29, 1.00		9.4	3.58	0.81, 15.9		10.7	8.37	0.92, 76.6	

Child's gender					0.2				0.008				0.2
Male	154	27.9	1.00			3.9	1.00			11.0	1.00		
Female	151	35.1	1.40	0.83, 2.34		11.9	3.34	1.40, 7.97		6.0	0.51	0.16, 1.61	

Mother's education					0.6				0.4				0.1
None	183	32.8	1.00			6.6	1.00			6.0	1.00		
Any	122	29.5	0.86	0.45, 1.63		9.8	1.55	0.56, 4.33		12.3	2.19	0.79, 6.06	

Mother's age					0.1				0.6				1.0
< 20 years	56	26.8	1.00			10.7	1.00			10.7	1.00		
20-29 years	120	38.3	1.70	0.74, 3.90		5.8	0.52	0.17, 1.56		8.3	0.76	0.11, 5.40	
30-39 years	93	31.2	1.24	0.47, 3.23		8.6	0.78	0.28, 2.21		7.5	0.68	0.10, 4.42	
> 40 years	36	16.7	0.55	0.11, 2.81		8.3	0.76	0.11, 5.14		8.3	0.76	0.11, 5.21	

No. wives in HH					0.6				0.05				0.4
One or less	208	30.3	1.00			9.1	1.00			9.6	1.00		
Two or more	97	34.0	1.19	0.65, 2.17		5.2	0.54	0.29, 1.00		6.2	0.62	0.21, 1.86	

Household size					0.4				0.1				0.05
< 10	112	36.6	1.00			12.5	1.00			2.7	1.00		
10-14	129	31.0	0.78	0.38, 1.61		7.0	0.53	0.23, 1.22		11.6	4.78	1.29, 17.7	
≥ 15	64	23.4	0.53	0.21, 1.31		1.6	0.11	0.01, 0.96		12.5	5.19	1.14, 23.6	

No. U5s with fever in HH					0.6				0.2				0.2
One	183	32.8	1.00			9.8	1.00			6.6	1.00		
Two or more	122	29.5	0.86	0.47, 1.56		4.9	0.47	0.16, 1.38		11.5	1.85	0.65, 5.24	

Socioeconomic status*					0.05				0.6				1.0
Lowest	98	25.5	1.00			7.1	1.00			7.1	1.00		
Middle	106	28.3	1.15	0.65, 2.04		6.6	0.92	0.23, 3.68		6.6	0.92	0.33, 2.55	
Highest	95	42.1	2.12	1.15, 3.90		10.5	1.53	0.42, 5.45		7.4	1.03	0.30, 3.57	

Seen malaria information in last year					0.6				0.1				0.3
No	127	33.1	1.00			4.7	1.00			11.0	1.00		
Yes	178	30.3	0.88	0.51, 1.54		10.1	2.27	0.84, 6.13		6.7	0.58	0.20, 1.68	

* 6 missing values for socioeconomic status

Not all variables remained associated when included in multivariable models (Table 3). For the public sector, child's age and socioeconomic status of the household remained predictors in the adjusted model. Older children (< 1 year olds: referent; 1-4 year olds: OR: 0.53; 95%CI: 0.28, 1.00; p = 0.05) and those of lower socioeconomic status (lowest tertile: referent; middle tertile: OR: 1.13; 95%CI: 0.64, 1.99; highest tertile: OR: 2.14; 95%CI: 1.17, 3.91; p = 0.04) were less likely to seek care from a public sector health facility when they have fever. The adjusted likelihood of a child with fever being taken to a community-based delivery point for first advice or treatment was greater for female children (OR: 3.53; 95%CI: 1.47, 8.45; p = 0.006). The adjusted likelihood of a febrile child seeking first treatment from a retail outlet increased with household size (<10 members: referent; 10-14 members: OR: 4.38; 95%CI: 1.17, 16.4; 15 or more members: OR: 4.69; 95%CI: 1.03, 21.3; p = 0.07)

**Table 3:** Multivariable logistic regression models of the odds for seeking any advice or treatment for a child under five years with fever from the public sector, community sector or retail sector (Note: multivariable regression not conducted for "any source" as only one variable met the inclusion criteria of a p-value < 0.1).

First seek treatment in public sector				First seek treatment in community sector				First seek treatment in retail sector			
**Variable**	**OR**	**95% CI**	**P value**	**Variable**	**OR**	**95% CI**	**P value**	**Variable**	**OR**	**95% CI**	**P value**

Child's age			0.05	Child's age			0.07	Child's age			0.08
< 1 year	1.00			< 1 year	1.00			< 1 year	1.00		
1-4 years	0.53	0.28, 1.00		1-4 year	3.53	0.90, 17.7		1-4 years	7.42	0.79, 69.6	

Socioeconomic status*			0.04	Gender			0.006	Household size			0.07
				Male	1.00						
Lowest	1.00			Female	3.53	1.47,8.45		< 10	1.00		
Middle	1.13	0.64, 1.99						10-14	4.38	1.17, 16.4	
Highest	2.14	1.17, 3.91						≥ 15	4.69	1.03, 21.3	

				No. wives			0.08				
				≤ 1	1.00						
				≥ 2	0.56	0.29, 1.07					

### Treatment received according to place first advice or treatment sought

A greater proportion of children with fever in the previous two weeks received an anti-malarial, if they first sought treatment from a community-based delivery point (62.5%; n = 15). This was more than twice the proportion of those who received an anti-malarial if they first visited a public sector health post or health centre (29.5% and 23.5%, respectively) (Table 4). Univariable logistic regression supports this finding with children first taken to a community-based service being approximately four times more likely to receive an anti-malarial than those first taken to a public health facility (public sector health facility: referent; community-based delivery point: OR: 4.20; 95%CI: 1.49, 11.80; retail sector: OR: 0.46; 95%CI: 0.11, 1.88; p = 0.01).

**Table 4:** Treatment given to children under five years with fever, according to place where first advice/treatment sought.

Place where advice/treatment FIRST sought	Treatment given							
	
	Any anti- malarial (AM)		AS-AQ		Antibiotic (AB)		Other (not an AM or AB)	
	
	%	n	%	n	%	n	%	n
Health hut/ASC/RC	62.5	15	45.8	11	0	0	37.5	9

Health post	29.5	23	19.2	15	15.4	12	48.7	38

Health centre	23.5	4	5.9	1	35.3	6	41.2	7

Retail sector	15.4	4	3.8	1	7.7	2	80.8	21

No antibiotics were given to children first seeking care from a health hut, which supports practice of the current national policy that antibiotics cannot be prescribed at the community level unless the community health workers are appropriately trained and supervised. The proportion of children seeking care in the public sector and receiving an antibiotic is higher (15.4% and 35.3% of those first seeking care at a health post or health centre, respectively).

Most of the 'other' treatments (i.e. neither an anti-malarial nor an antibiotic) given to febrile children were paracetamol, regardless of the source of first treatment/advice (community-level: 7/9; formal public health facility: 30/45; retail sector: 19/21). Approximately half of children (46/94) receiving an anti-malarial or antibiotic also received paracetamol. Overall, 1.6% (5) children received both an anti-malarial and an antibiotic. Nine percent of the children taken to a health post as the first source of treatment or advice did not ultimately receive any treatment; this compares with 40% of those initially taken to a traditional healer. All children who were first taken to a health hut or health centre received some kind of treatment.

The proportion of febrile children taking an ACT within 48 hours was 6.2% (19) from any source, 3.0% (9) from a formal public provider, 3.0% (9) from a community source and 0.3% (1) from the retail sector. Only 1.6% (5) also took the correct dose, and 1.3% (4) for the correct duration forming the complete indicator for prompt and effective treatment, all of whom were children first taken to a community provider.

### Treatment process analysis

**Figure 1:**
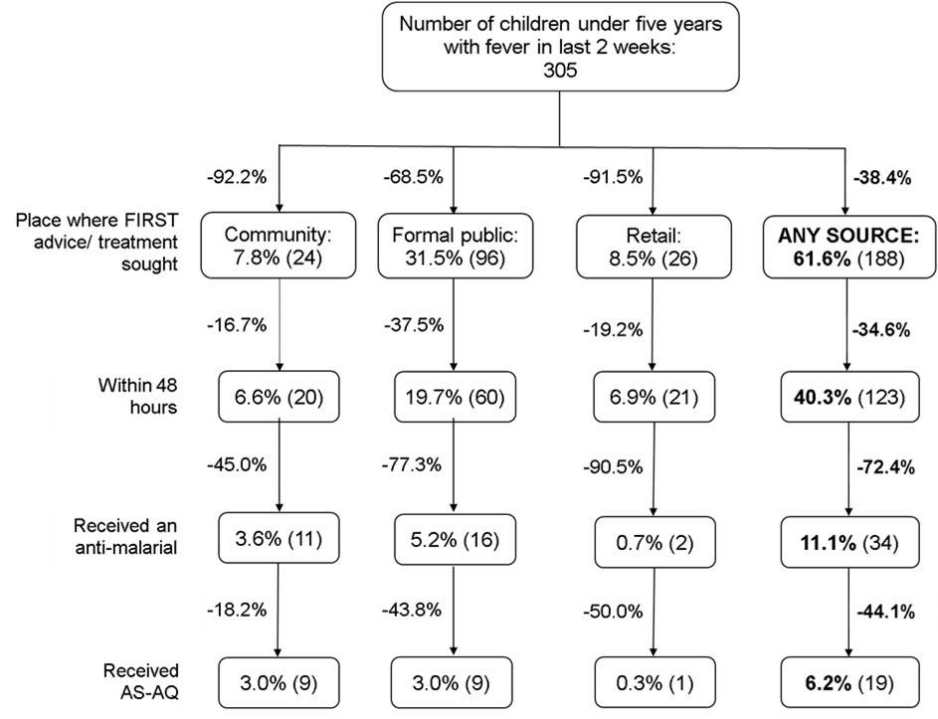
**Treatment with an anti-malarial by source of first advice or treatment. **Figures within boxes are proportions of all febrile children; percentages between boxes indicate the proportion of children that exit at each step in the pathway. 'Any source' indicates the total for each step, not stratified by provider.

By categorising the treatment process into key steps it is possible to visualise the proportion of children progressing through each step and to investigate at which step the greatest number of children exit the process required to receive effective treatment. This analysis can be further stratified by the sector or treatment delivery point of first seeking treatment (Figure 1). Overall, the greatest proportional loss occurs after treatment has been sought from any source; over 70% of children seeking treatment do not go on to receive an anti-malarial. Amongst those children that are given an anti-malarial just over half (55.9%) receive an ACT.

Simple modelling of changes at various steps in the treatment process, based on the empirical data shown in Figure 1, suggests that increasing the proportion of children seeking care at community based treatment delivery points (health huts) would be a relatively effective way of increasing the proportion of febrile children receiving effective treatment. Increasing the proportion of children seeking treatment from a community delivery point (7.8%) to the level of current treatment-seeking from the formal public sector (31.5%) increases the proportion ultimately receiving treatment with an ACT from 2.9% to 11.8% (Table 5). Increasing the proportion of febrile children seeking treatment from a community delivery point to the current percentage seeking treatment from any provider (61.6%) increases treatment with an ACT to 23.1%.

**Table 5:** Model to show how varying the proportion of febrile children passing through each step in the treatment pathway for those seeking care from a community delivery point influences the ultimate proportion that receive an ACT (unadjusted for RDT use).

	Actual data			Same proportion accessing as currently go to public sector			Same proportion accessing as currently go to any provider		
**Community delivery point**	**N**	**%**	**Cum.%**	**N**	**%**	**Cum.%**	**N**	**%**	**Cum.%**

1. U5s with fever in last 2 weeks	305	100	100	305	100	100	305	100	100

2. Sought any advice or treatment	24	7.8	7.8	96	31.5	31.5	188	61.6	61.6

3. Within 48 hours	20	83.3	6.5	80	83.3	26.2	157	83.3	51.3

4. Received an AM	11	55.0	3.6	44	55.0	14.4	86	55.0	28.2

5. Received an ACT	9	81.8	**2.9**	36	81.8	**11.8**	70	81.8	**23.1**

However, the improvements seen in the outcome of receiving prompt effective treatment with an ACT remain modest if increasing the proportion of children successfully completing the process focuses on just one step in the pathway. Indeed, even when every step in the process was increased by 20%, the overall proportion of febrile children who received an ACT from any source was still only 25.2% (see Additional file 1 for full results of this modelling process).

### Modelling the inclusion of RDT-confirmed diagnosis

Based on the PNLP data for 2008, we assume that 78% of febrile children taken to a formal public sector health provider were tested with an RDT, and 60% of these were positive for malaria parasites. Applying these data to the survey findings and assuming that the 22% that do not receive an RDT have strong alternative clinical diagnoses, the proportion of children appropriately receiving an anti-malarial promptly from the public sector would rise from 16.7% (16/96) to 35.6% (16/45) and the proportion appropriately receiving an ACT from the public sector would rise from 9.4% (9/96) to 20.0% (9/45) when RDT confirmation was included. Because the role of diagnosis in the definition of "effective" treatment is different across sectors, it is not possible to construct an overall indicator for prompt and effective treatment of the original cohort of 305 febrile children if public sector RDTs are included since the denominator for the public sector then becomes different to that for the community and retail sectors.

## Discussion

It is clear that large proportions of children exit from each step in the treatment pathway, and that a variety of interventions will be required to increase the proportion of febrile children with malaria receiving prompt and effective treatment with an ACT in the study area. Almost 40% of children with fever are not taken for any advice or treatment. This may be because the fever was only of short duration and the child quickly recovered, for example due to self-medication with drugs already in the home and/or other first aid measures such as tepid sponging. Nevertheless, the result still highlights the importance of health promotion to improve awareness of malaria symptoms and the importance of seeking prompt treatment if initial home measures such as tepid sponging do not work. The proportion of children not seeking any treatment seems high in relation to other studies [21,22]; however it is similar to findings from Uganda [23], which also reported approximately 40% of children not seeking any care for fever. More investigation is clearly needed to understand initial treatment practices in the home, the reasons for not seeking treatment outside the home, and to follow up on health outcomes including estimation of the proportion of fevers that are self-limiting.

Perhaps the starkest finding from this process analysis of the treatment pathway was that over 70% of febrile children taken to any provider did not receive an anti-malarial. Given the national policy for confirmed diagnosis in formal public health facilities, it is not unexpected that a lower proportion of febrile children received an anti-malarial when taken to a public health facility rather than a community delivery point. However, assuming that 47% of fevers are malaria (78% febrile children receive an RDT and of these 60% are positive), one would expect 47% of febrile children to receive an ACT in the public sector whilst our findings show that only 26.7% of those promptly seeking treatment in the public sector received any anti-malarial and 15.0% an ACT. The proportion of those promptly seeking treatment for a febrile child at any provider and not being given an appropriate anti-malarial (i.e. AS-AQ) is 84.6%. The not uncommon occurrence of children being given anti-malarials other than ACT (including chloroquine) in the public sector is an indication that improvements are still necessary. The results of the present study support those of the 2008 Senegal Malaria Indicator Survey, which found that only 2.2% of children in the Tambacounda region with fever in the previous two weeks received an ACT on the same or next day [24].

Effective treatment of malaria does not stop at prescription of an ACT to patients with a confirmed diagnosis; the caretaker of the child also needs to access the prescribed medication and fully adhere to the complete treatment regimen making sure that the child takes the correct dose and for the correct number of days. In addition to including an indication of promptness of treatment-seeking (within 48 hours of the child first showing signs of fever), these elements make up the full RBM indicator of prompt and effective treatment of malaria [1]. In this study, only 1.3% of children with fever received an ACT within 48 hours of presenting with fever, which was reportedly taken at the correct dose and for the correct duration.

The exiting of children from steps in the treatment pathway seen in this study is very similar to findings from Uganda; Nsungwa-Sabiiti *et al *found that in a population of 260 children with fever, 87% received any treatment, 44% within 24 hours, 26% with an appropriate anti-malarial and only 7% at correct dose and duration [25]. This emphasizes the need to target both users and providers if improvements in the prompt and effective treatment of malaria in children are to be achieved. Qualitative work is now needed to understand the reasons for the observed losses at each step in the treatment pathway in order to design appropriate interventions.

Studies investigating how the use of RDTs in routine health services influence clinical decisions have shown mixed results. For example, research in Tanzania and Burkina Faso found that many health providers continue to prescribe anti-malarials to patients with a negative RDT result [26,27], whereas this practice was much less frequent in studies in Kenya and Zanzibar [28,29]. Modelling of the impact of RDT-confirmed diagnosis did not incorporate interpretation of the result into the treatment received by the febrile child, meaning that some of those given an anti-malarial may have been negative and some of those not given an anti-malarial may have been positive which would have influenced the proportion of febrile children being appropriately treated. RDTs are likely to be less cost effective if the results are not used properly, particularly in areas with falling malaria transmission such as Senegal [12] and where treatment is with more expensive ACTs [30,31]. A key intervention in addition to increasing the use of RDTs in the public sector is, therefore, to encourage appropriate treatment of positive results with an anti-malarial (more importantly, an ACT) and negative results with a suitable alternative treatment, for example an antibiotic or antipyretic.

Interestingly, the proportion of febrile children taken to a health centre that were given an antibiotic supports that differential diagnosis based on a confirmed RDT may have been appropriately implemented since almost 40% of children received an antibiotic (approximately the proportion that would have been expected to receive a negative RDT). Overall, 1.6% of children received both an anti-malarial and an antibiotic. However, at the health post level only 15% of febrile children received an antibiotic. It is possible that the prescription of ACT was low in the public sector due to problems with stock-outs, known to have adverse effects on appropriate treatment of malaria in other countries after changing their national malaria drug policy [32]. Alternatively, it may have been because of health worker reluctance to prescribe AS-AQ due to perceived or observed side effects of amodiaquine which have previously been found in Senegal [33]. Household surveys are currently the standard method used to assess the RBM malaria treatment indicator; however, to truly understand the provider-side factors such as stock and quality of care that influence the ultimate treatment received and adhered to by children with malaria, it is necessary to also conduct concurrent health facility and health worker surveys.

In addition to the existing use of RDTs in public health facilities, they are also presently being rolled out for use in the diagnosis and treatment of malaria at the community level in Senegal. Community health workers (ASCs) will be supplied with RDTs and trained in their use. Use of RDTs by community volunteers has been shown to be feasible in other settings [7,8]; however, concerns with appropriate use of the tests and correct interpretation of results mean that close monitoring of the new system will be important. Similarly, the inclusion of a confirmed malaria diagnosis as a specific step in the treatment pathway, regardless of source of treatment, will be important in assessing effective treatment of febrile children in the intervention area in future surveys.

Another interesting, if not surprising, finding of this study is that there is minimal activity in the retail sector in relation to ACT; although 8.5% of febrile children were taken to a retail outlet, only 3.8% of these received an ACT.

The predictors for a child with fever seeking treatment vary depending on the type of provider that they are initially taken to. For example, access to the public sector increases with higher socioeconomic status. However, socioeconomic status is not a predictor of accessing community-based delivery points. This supports data from other studies that suggest providing ACT through community-based systems such as health huts may be an appropriate way to increase equity of access to effective malaria treatment [34]. However, there is still debate as to whether community health workers are able to increase socioeconomic equity in the coverage of interventions as effectively as they improve geographic equity, especially as some level of payment is usually involved [35,36]. Finding that carers of febrile children were seeking treatment from the community sector in areas with non-functional health huts may have been due to a small number of health huts being rehabilitated and receiving their ACT supplies whilst the survey was underway, or to people travelling large distances to access functioning health huts.

The sample size is a limitation of this survey, resulting in wide 95% confidence intervals. This is due to the decreasing numbers of children progressing to each step of the treatment pathway, particularly when they are stratified by provider and categorized determinant. Future studies wishing to assess the different steps in the treatment pathway, particularly when including stratification by type of provider, should allow a sufficiently large sample size to account for the decreasing numbers of children remaining in the evaluable sample as the pathway progresses.

It is important to note that there may be some inaccuracies in the names and types of drugs that the caretakers reported giving to their children [37], particularly due to problems with recall of an event that occurred up to two weeks ago. Although we tried to limit this source of bias by providing the interviewers with a photo board for anti-malarials available in the region, non anti-malarials were not included. It is difficult to predict however, whether this would mean anti-malarials or non anti-malarials would have a greater chance of being reported by caretakers.

Despite the sample size and other limitations, it is clear that large proportions of children exit from each step in the treatment pathway, and that intervention is required at multiple steps to increase the proportion of febrile children with malaria receiving prompt and effective treatment in the study area.

## Conclusion

Progress towards achieving the RBM goal of 80% of malaria patients being diagnosed and treated with effective anti-malarial medicines (ACT) within one day of the onset of illness is slow. In order to understand the reasons for this and to generate improvements, it is necessary to identify the steps within the treatment pathway where the greatest proportion of children exit from the process of receiving effective treatment. This will enable the identification of the steps where interventions can or should be targeted to achieve the greatest impact. Limited impact will be achieved if interventions only target one step in the pathway. In the Tambacounda region of Senegal, the most important targets for interventions are increasing initial care seeking for fever, improving uptake of RDTs at the public and community levels and correctly treating parasite positive patients with an ACT. There is also a need for complementary qualitative work to investigate in more detail why these processes are not occurring effectively so that the most appropriate interventions can be designed. It is important that this diagnosis of the treatment pathway is further broken down by provider type since the public, community-based and retail sectors behave differently but are all important if the RBM targets for prompt and effective treatment of malaria are to be achieved.

## Abbreviations

ACT: artemisinin combination therapy; AS-AQ: artesunate-amodiaquine; ASC: *agent de santé communautaire *[community health worker]; CI: confidence interval; EPI: Expanded Programme on Immunisation; OR: odds ratio; PNLP: *Programme National de Lutte Contre le Paludisme *[National Malaria Control Programme]; RC: *relais communautaire *[community health volunteer]

## Competing interests

The authors declare that they have no competing interests.

## Authors' contributions

LS, JB, JW, and CJ devised the study design and objectives. LG, AH, LS, JW, JB, CJ, BG and RD contributed to data collection, analysis and interpretation. LS did the analysis and wrote the first draft of the manuscript. All authors read, commented on and approved the final manuscript.

## Supplementary Material

Additional file 1 Model to show how varying the proportion of febrile children passing through each step in the treatment pathway (from any provider) influences the ultimate proportion that receives an ACT (unadjusted for RDT use). Click here for file
