# Supplementary material for: From fever to anti-malarial: the treatment-seeking process in rural Senegal
Source: Malar J. 2010 Nov 22;9:333. doi: 10.1186/1475-2875-9-333 (PMC3000420; doi:10.1186/1475-2875-9-333)
Supplement: Additional file 1 — Model to show how varying the proportion of febrile children passing through each step in the treatment pathway (from any provider) influences the ultimate proportion that receives an ACT (unadjusted for RDT use). [file 1475-2875-9-333-S1.DOC]

**ADDITIONAL FILE 1**

Model to show how varying the proportion of febrile children passing through each step in the treatment pathway (from any provider) influences the ultimate proportion that receives an ACT (unadjusted for RDT use).

|  | Actual data | | | 20% increase in step 2 | | | 20% increase in step 3 | | | 20% increase in step 4 | | | 20% increase in step 5 | | | 20% increase in steps 2-5 | | |
| --- | --- | --- | --- | --- | --- | --- | --- | --- | --- | --- | --- | --- | --- | --- | --- | --- | --- | --- |
| Any Source | N | % | Cum. % | N | % | Cum. % | N | % | Cum. % | N | % | Cum. % | N | % | Cum. % | N | % | Cum. % |
| 1. U5s with fever in last 2 weeks | 305 | 100 | 100 | 305 | 100 | 100 | 305 | 100 | 100 | 305 | 100 | 100 | 305 | 100 | 100 | 305 | 100 | 100 |
| 2. Sought any advice or treatment | 188 | 61.6 | 61.6 | 249 | 81.6 | 81.6 | 188 | 61.6 | 61.6 | 188 | 61.6 | 61.6 | 188 | 61.6 | 61.6 | 249 | 81.6 | 81.6 |
| 3. Within 48 hours | 123 | 65.4 | 40.3 | 163 | 65.4 | 53.4 | 160 | 85.4 | 52.6 | 123 | 65.4 | 40.3 | 123 | 65.4 | 40.3 | 213 | 85.4 | 69.7 |
| 4. Received an AM | 34 | 27.6 | 11.1 | 45 | 27.6 | 14.7 | 44 | 27.6 | 14.5 | 58 | 47.6 | 19.2 | 34 | 27.6 | 11.1 | 101 | 47.6 | 33.2 |
| 5. Received an ACT | 19 | 55.9 | **6.2** | 25 | 55.9 | **8.2** | 25 | 55.9 | **8.1** | 33 | 55.9 | **10.7** | 26 | 75.9 | **8.4** | 77 | 75.9 | **25.2** |
